# Applications of Fluorogens with Rotor Structures in Solar Cells

**DOI:** 10.3390/molecules22060897

**Published:** 2017-05-29

**Authors:** Kok-Haw Ong, Bin Liu

**Affiliations:** Institute of Materials Research & Engineering, Agency for Science, Technology and Research (A*STAR), 2 Fusionopolis Way, Innovis, #08-03, Singapore 138634, Singapore; ongkh@imre.a-star.edu.sg

**Keywords:** aggregation-induced emission, solar cell, fluorogens, rotor structures

## Abstract

Solar cells are devices that convert light energy into electricity. To drive greater adoption of solar cell technologies, higher cell efficiencies and reductions in manufacturing cost are necessary. Fluorogens containing rotor structures may be helpful in addressing some of these challenges due to their unique twisted structures and photophysics. In this review, we discuss the applications of rotor-containing molecules as dyes for luminescent down-shifting layers and luminescent solar concentrators, where their aggregation-induced emission properties and large Stokes shifts are highly desirable. We also discuss the applications of molecules containing rotors in third-generation solar cell technologies, namely dye-sensitized solar cells and organic photovoltaics, where the twisted 3-dimensional rotor structures are used primarily for aggregation control. Finally, we discuss perspectives on the future role of molecules containing rotor structures in solar cell technologies.

## 1. Introduction

A solar cell or photovoltaic cell is an electrical device that converts the energy of light into electricity. In recent years, there has been a significant acceleration in the adoption of solar cell technologies, with photovoltaic installations experiencing a compound annual growth rate of 42% between 2000 and 2015. This has been driven by the need for renewable, environmentally friendly energy sources, as well as cost reductions which have enabled solar power to be cost-competitive vis-à-vis power generated from fossil fuels [1]. To date, the majority of solar cell installations are based on first-generation technologies, which are wafer-based cells primarily manufactured from mono-crystalline and poly-crystalline silicon [2,3]. Silicon-based cells generally show high efficiency, but suffer from drawbacks such as high energy consumption during production and a relatively thick light-absorbing layer required for efficient light harvesting. Second-generation thin-film-based solar cell technologies such as cadmium telluride (CdTe) and CuInGaSe_2_ (CIGS) cells and third generation emerging technologies based on organic materials such as dye-sensitisized solar cells (DSSCs) and organic photovoltaic cells (OPVs) have the potential to overcome these drawbacks by enabling flexible and lightweight modules. Different strategies, such as luminescent solar concentrators (LSCs) can also improve the effectiveness of light harvesting by collecting photons over a large area and channeling them to a smaller solar cell [4]. However, for each of these technologies, further improvements are necessary to achieve higher efficiencies and reduce costs. 

CdTe is considered the leading second generation solar cell technology, comprising more than half of the production of thin-film cells [5]. One of the challenges for CdTe cells is that their spectral response in the sub-500 nm region is typically poor due to the parasitic absorption by the cadmium sulfide (CdS) layer in such cells. This results in reduced photocurrents, which limit the efficiency of the modules. Luminescent down-shifting (LDS) layers are thus required to mitigate these losses [6]. These layers incorporate luminescent dyes that absorb short-wavelength light and emit photons at longer wavelengths which better match the spectral response of the solar cells. Commercially-available dyes, however, have limited efficacy when used with state-of-the-art CdTe cells due to parasitic absorption. Commercial dyes also have a tendency to π-stack, which reduces their fluorescence quantum efficiency and limits the concentration of the dyes within the LDS layer. Furthermore, their limited Stokes shifts lead to re-absorption losses. As such, dyes with better spectral matching, reduced π-stacking tendency, and larger Stokes shifts are desired. In a similar manner, dyes with large Stokes shifts and reduced tendency to π-stack are desirable for LSC applications. 

For the third-generation cells such as DSSCs and OPVs, improvements in materials and device structures have driven efficiencies to above 10% [7]. However, the solar-active materials used, such as metal-based dyes in DSSCs and fullerene-based acceptors in OPVs, are expensive and cheaper alternatives for these are desired. However, metal-free dyes and non-fullerene acceptors are currently unable to match the performance of their more-established counterparts [8,9,10]. An important factor in the design of both types of molecules is the control of aggregation. In the context of DSSC dyes, dye aggregation may lead to increased back-electron transfer, which reduces the photocurrent generated. For OPVs, aggregation of the acceptor molecules results in large acceptor domains, leading to reduced photocurrent as excitons generated too far away from the donor-acceptor interface recombine without being charge-separated at the interface.

The unique properties of rotor structures have the potential to address the issues elucidated above. The concept of aggregation-induced emission (AIE) was first reported in 2001 by Tang and co-workers [11]. Unlike most luminogens which exhibit reduced or no emission at high concentrations, a phenomenon which has been termed aggregation-caused quenching (ACQ), fluorophores with AIE properties (AIEgens) have low emissions in dilute solutions but become highly emissive when aggregates are formed, such as in concentrated solutions or in the solid state. Many AIEgens have also been reported to have large Stokes shifts. Since the first report of AIE in hexaphenylsilole molecules, a wide variety of molecules with AIE and aggregation-induced enhanced emission (AIEE) properties have been synthesized and characterized. The unique photophysical properties of AIEgens and AIEE systems have been studied extensively, and applications such as biological probes, chemical sensing, and optoelectronic devices have been demonstrated [12,13]. In recent years, new synthetic methodologies have also made novel AIE and AIEE macromolecules and polymers possible [14,15].

**Figure 1:**
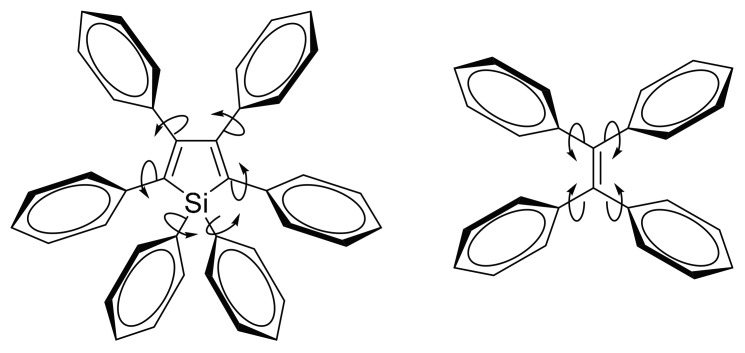
Structure of hexaphenylsilole (HPS) and tetraphenylethylene (TPE), illustrating the free rotation of the phenyl rings in solution.

A major class of AIEgens comprises three-dimensional propeller-shaped molecules such as hexaphenylsilole (HPS) and tetraphenylethylene (TPE) [12,16]. Due to steric repulsion between the phenyl rings, these molecules are highly twisted and experience very little π-stacking in the solid state. Rather, the crystals are held together by multiple C−H···π interactions between adjacent molecules. In dilute solutions, the peripheral phenyl rings act as rotors—that is, they are free to rotate around the axes of the single bonds (Figure 1)—thus dissipating excitonic energy in a non-radiative manner. Upon aggregate formation, the C−H···π interactions restrict the rotation of the phenyl rings, thus suppressing the non-radiative pathway and enabling the radiative pathway. The incorporation of rotors in dye molecules may therefore lead to more effective dyes that have large Stokes shifts and have enhanced emission at high concentrations instead of being quenched. Furthermore, although their emission characteristics are not required in DSSC dyes and non-fullerene acceptors for OPVs, the incorporation of rotor structures in these molecules can help to suppress π-stacking and aid in aggregation control. 

While the application of AIEgens in solar cell technologies has been covered as part of comprehensive reviews on AIE [12], other molecules with related rotor structures, but which are not necessarily fluorescent, have yet to be covered. In this mini review, we summarize the applications of the typical rotor-containing molecules in various solar cell technologies. Firstly, we discuss the use of AIEgens in LDS layers for second generation (thin-film) solar cells. We then discuss the use of AIEgens in LSC devices. We will then move on to the applications of molecules containing rotor structures such as HPS, TPE, and triphenylethylene in third-generation solar cell technologies, namely DSSCs and OPVs. Finally, we discuss perspectives on the future role of molecules containing rotor structures in solar cell technologies.

## 2. Luminescent Down-Shifting Layers for Second Generation CdTe Cells

**Figure 2:**
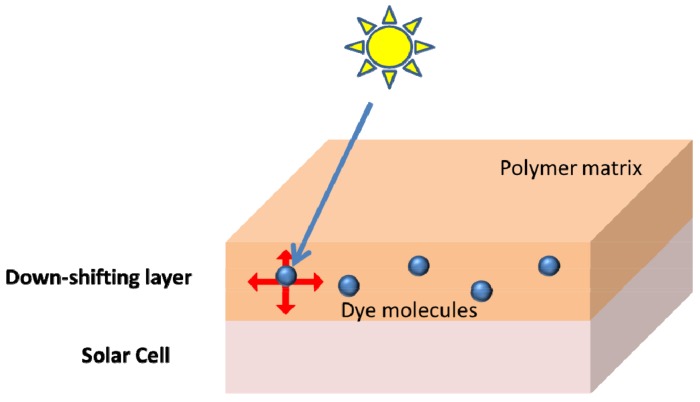
Structure of a typical luminescent down-shifting layer.

Solar cells based on cadmium telluride (CdTe) are promising candidates for next generation solar cells because of their relatively low manufacturing cost compared with traditional silicon-based cells. However, CdTe cells typically have poor spectral responses in the sub-500 nm region, corresponding to the absorption of the cadmium sulfide layer in the cell structure, which leads to losses. Apart from the optimization of the cell structure, another method of reducing such losses is to incorporate a luminescent down-shifting (LDS) layer on the front surface of the cell. This is typically composed of luminescent molecules embedded within a polymer film, which absorb short wavelength photons and emit long wavelength photons, thereby altering the spectrum of light which reaches the solar cell (Figure 2) [6]. Luminescent dyes such as BASF Lumogen F dye Yellow 083 (Y083) have been successfully used to significantly increase the *J_sc_* of the cells. However, due to improvements in the cell structure over time, the spectral responses of the cells at short wavelengths have increased, resulting in increased parasitic effects when LDS layers based on Y083 are used. The planar perylene molecules in Y083 also give it a tendency to π-stack, resulting in reduced fluorescence quantum yield (*Φ_f_*) in the solid state.

**Figure 3:**
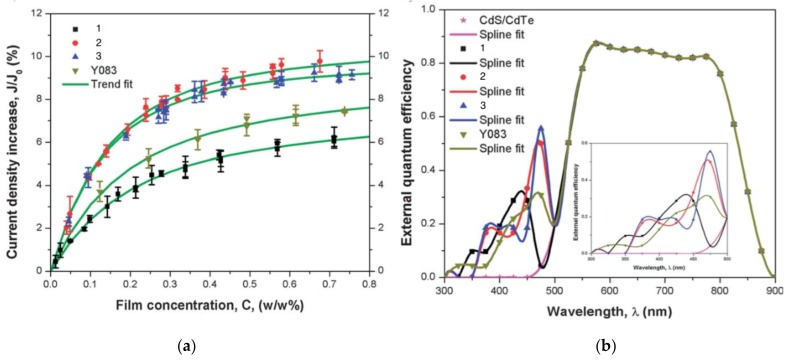
(**a**) The increase of short circuit current density (*J_sc_*) vs. the different concentrations of the LDS film placed on the CdTe solar cell surface for fluorophores 1–3 and Y083; (**b**) The enhanced spectral response at short-wavelengths after the LDS film was placed on the CdTe solar cell surface for fluorophores 1–3 and Y083. (reprinted with permission from ref. [17], Copyright 2013 Royal Society of Chemistry).

In this regard, Dong and co-workers designed a series of fluorophores based on TPE and malonitrile (**1**–**3**) (Table 1) [17]. In the presence of the strong electron-withdrawing malonitrile group, the TPE moieties act as an electron donor. The donor-acceptor interactions result in the fluorophores having emission maxima (λ_em_) of around 550 nm. Compared with Y083, fluorophores **1**–**3** have significantly larger Stokes shifts (*Δ*λ) of 129 to 146 nm. The fluorophores were confirmed to have AIE properties, as they have low fluorescence intensities in good solvents with low water fractions (*f_w_*), while the fluorescence intensities increase significantly when *f_w_* is increased to over 60%. When applied in LDS devices, the molecules delivered significant improvements in *J_sc_* of 6 to 10%. Notably, the performances of **2** and **3** were better than the control Y083 devices, which delivered an increase in *J_sc_* (*ΔJ_sc_*) of 8% (Figure 3a). The improved performance could be attributed to the improved absorption of short-wavelength photons by the fluorophores as reflected in the improved spectral response (Figure 3b), as well as the enlarged Stokes shift and high fluorescent quantum yields.

The same group also reported fluorophores based on TPE and dicyanoethylene (**4**–**6**) [18]. Photophysical studies of **4**–**6** showed that the Stokes shifts of the molecules in the solid state were between 139 and 170 nm, far higher than the 64 nm of Y083. All three fluorophores exhibited AIE characteristics, and **4** and **5** also exhibited larger solid-state fluorescent quantum yields than Y083. The application of PMMA films doped with these fluorophores as LDS layers on CdTe cells enhanced the *J_sc_* of the cells by as much as 5.69% for small area cells and 8.88% for large area cells, which were superior to that obtained using Y083 (3.28% and 4.01%, respectively). The high performance of the fluorophores could be attributed to a combination of lower parasitic absorption losses, better spectral matching of the emission with the spectral response of the CdTe cell, and higher solid-state fluorescent quantum yield.

The commercially available Y083 and fluorophores **1**–**6** all contain cyano (CN) groups, which meant that toxic precursors are inevitably used during the synthesis of the fluorophores. Dong and co-workers thus designed a TPE-perylene fluorophore with no CN groups (**7**), which would be more environmentally friendly [19]. Fluorophore **7** exhibited a much higher fluorescence quantum yield and fluorescence lifetime when dispersed in a polymethylmethacrylate (PMMA) film compared with its THF solution, and this was attributed to the intramolecular energy transfer and restriction of intramolecular rotation of the fluorophore in the solid state. Based on theoretical calculations, LDS films made from **7** would be able to increase the *J_sc_* of CdTe cells by up to 2.95%. In experimental cells, the *J_sc_* of the cells was increased by 3.30 ± 0.31%, which was close to the predicted value, and was comparable with the performance of Y083.

## 3. Luminescent Solar Concentrators

**Figure 4:**
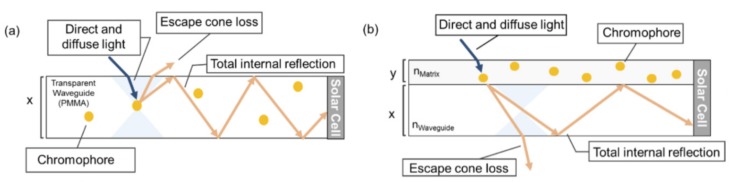
Conventional structures for luminescent solar concentrator (LSC) applications: (**a**) millimeter-thick waveguides infused with fluorescent dyes; (**b**) micron-thick thin layer matrices cast on top of a waveguide. (reprinted with permission from ref. [22], Copyright 2014 Nature Publishing Group).

Luminescent solar concentrators (LSCs) are devices used to improve the output of solar devices. Highly luminescent materials embedded within transparent substrates absorb light and produce emission which is then channeled to the solar cells using waveguides (Figure 4). The simplicity of these devices means that they can easily be integrated into building structures such as walls and windows [20].

The materials used in LSCs are closely related to those used in LDS. A key difference between these two technologies is that for LSCs, the solar cells are positioned at the edge of the fluorescent layer, while for LDS, the solar cells are in optical series with the fluorescent layer [21]. To date, laser dyes such as perylenes, coumarins, and rhodamines have been commonly used in LSCs [20]. These dyes suffer from drawbacks which limit their performance in LSCs. Firstly, their planar conjugated structures result in a tendency to form aggregates which are non-emissive. Secondly, they have small Stokes shifts, which cause losses due to reabsorption of emitted light. The concentration of the dyes therefore has to be limited, resulting in transmission losses. 

**Figure 5:**
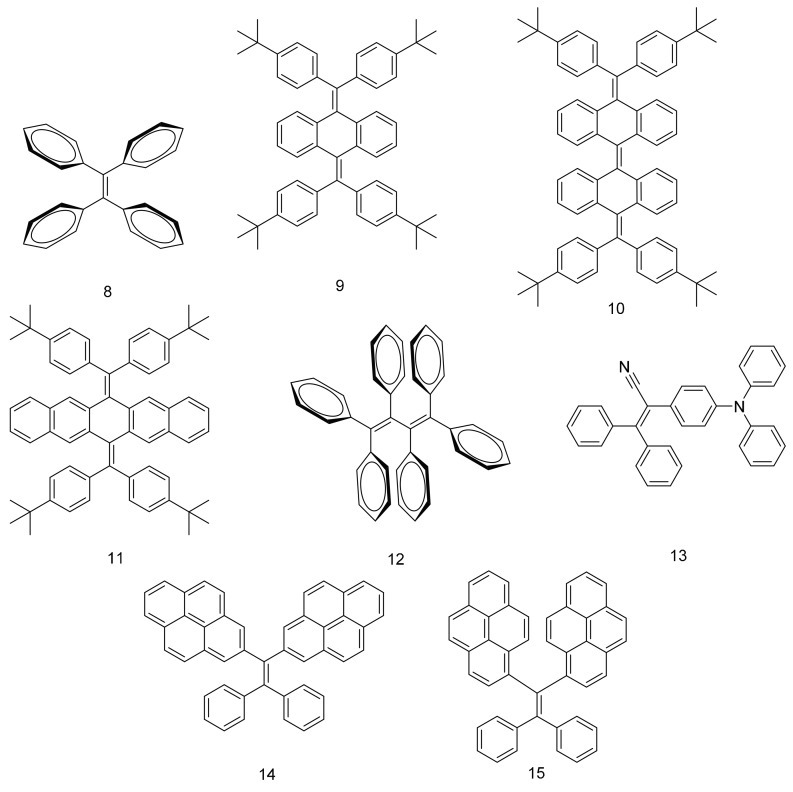
Structures of molecules **8**–**15** containing rotors in LSC applications.

To overcome these problems, Wong and co-workers explored the use of AIEgens in dye structures, using a combination of modeling and experimental studies [22]. Initial results on tetraphenyleneethylene** 8 **(Figure 5) revealed that it was promising for harvesting photons in the UV range. The optical edge efficiency (η_edge_) of an LSC fabricated using **8** with a geometric ratio (G) of 2.5 was 13.2%, which was close to the 13.4% predicted by simulations. Increasing G to 250 was calculated to improve the η_edge_ to as high as 26.7% with minimal reabsorption loss of 0.3%. However, the emission range was not ideal for LSCs that are coupled to high efficiency silicon or GaAs solar cells. The study was therefore expanded to include contorted or twisted polyaromatic hydrocarbons **9**–**12** in an attempt to expand the shift of the absorption and emission wavelengths; however increasing the number of aromatic rings was found to ineffective in achieving this aim. The fluorescence behavior of these molecules was investigated to confirm that they were AIE-active. The thin-film emission quantum yields of **9**–**11** were relatively low (between 4 and 10%), and were observed to decrease with increasing numbers of polyaromatic rings. This may be attributable to increased voids in the crystal lattice for the larger molecules, which allow greater freedom for the phenyl rings to rotate. The fully substituted butadiene (**12**) exhibited a more promising fluorescence quantum yield (31%), however it was still lower than the ethylene-based **8**. 

Another way to reduce reabsorption losses has been to use excitation energy transfer (EET) to induce a large Stokes shift. The EET approach utilizes two or more chromophores which may be covalently bound to a macromolecule or on separate dye molecules. The systems were designed in such a way that most of the emission is from the chromophore or dye that emits at the longest wavelength. However, covalently bound systems are typically difficult to synthesize and the fluorescence quantum yields are usually low, while dye aggregation and reabsorption losses are issues in non-covalent systems. To overcome this issue, Wong and co-workers utilized an AIE molecule, 2-(4-(diphenylamino)phenyl)-3,3-diphenylacrylonitrile (**13**) as the donor material, in combination with a low concentration of an acceptor dye with high emission quantum yield, 4-(dicyanomethylene)-2-*tert*-butyl-6-(1,1,7,7-tetramethyljulolidyl-9-enyl)-4*H*-pyran (DCJTB) [23]. Due to the AIE properties of **13**, it could be used in high concentrations to ensure efficient light harvesting and close proximity to the emission dye molecules—within the energy transfer critical radius—without experiencing the deleterious effect of concentration quenching. Its large Stokes shift also minimizes reabsorption losses. The short-circuit current of LSCs fabricated using the **13**:DCJTB system was significantly higher than that of the control DCJTB:PMMA system, showing the effectiveness of **13** in light harvesting.

**Figure 6:**
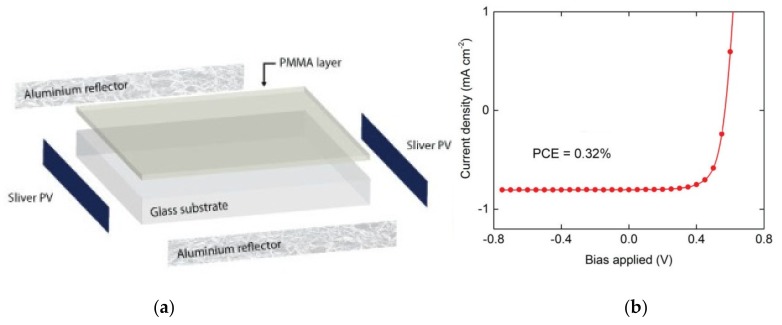
(**a**) Structure of the LDS device; (**b**) Current-voltage characteristics of a transparent planar concentrator device fabricated using 15. (reprinted with permission from ref. [4], Copyright 2017 American Chemical Society).

Wong and co-workers also investigated the properties of *gem*-substituted pyrene ethenes (**14**–**15**), which have a twisted structure to inhibit π-π stacking [24]. These molecules exhibited aggregation-enhanced emission (AEE). The twisted structure also resulted in large Stokes shifts, comparable to that of the pyrene excimer (1.06 eV). It was shown that the photophysical properties of these molecules depended strongly on the regioisomer structure, with **15** having much higher emission fluorescence quantum yields than **14** in both solution and thin films. **15** was applied in semitransparent planar concentrators and it was shown that its performance was similar to inorganic phosphors, while being more stable in an oxygen environment (Figure 6).

## 4. Dye-Sensitized Solar Cells (DSSCs)

**Figure 7:**
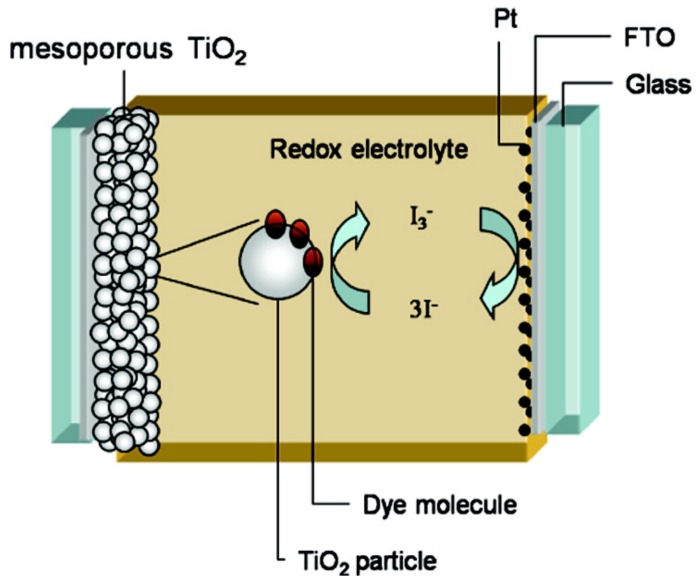
Schematic of a typical dye-sensitized solar cell (DSSC). (reprinted with permission from ref. [27], Copyright 2010 American Chemical Society).

Since the first report of DSSCs by O’Regan and Grätzel in 1991 [25], DSSCs have been widely studied due to their potential for low-cost manufacturing. A DSSC generally consists of a transparent electrode, a mesoporous titanium dioxide (TiO_2_) film on which a sensitizer is coated, a hole-transporting electrolyte, and a counter electrode (Figure 7). Incident light is absorbed by the sensitizer, followed by charge separation at the interface of the sensitizer and the TiO_2_. The holes are transferred to the electrolyte, which is then regenerated at the counter electrode by electrons from an external circuit [26].

DSSCs have traditionally been built upon dyes which are metal complexes. In recent years, there has been increasing interest in metal-free organic dyes, due to advantages such as potentially lower costs and the relative ease of tuning the dyes’ electrochemical and optical properties [9,26,28]. The control of dye aggregation on the titanium dioxide surface has been identified as an important factor in determining the efficiency of DSSCs based on metal-free organic dyes, as dye aggregation is believed to result in increased back-electron transfer which competes with the forward electron transfer to the electrolyte, thereby lowering the photocurrent [26]. In this regard, molecular rotors have been incorporated into metal-free DSSC dyes with promising results. 

An early example of this was reported by Horiuchi and co-workers in 2003 [29]. A series of indoline dyes was prepared, among which was a dye (**16**) containing a triphenylethylene group (Table 2). The extinction coefficient for **16** was 55,800 mol^−1^ cm^−1^ at 491 nm, which was higher than the 13,900 mol^−1^ cm^−1^ at 541 nm for the benchmark ruthenium-based N3 dye under the same conditions. The adsorption of **16** on TiO_2_ electrodes resulted in the broadening and red-shifting of the absorption peak, which was attributed to the formation of J-aggregates. When used in solar cells, a power conversion efficiency (PCE) of 6.1% was obtained, which was considered quite high for metal-free organic dyes. As a comparison, cells containing a dye with a naphthalene group instead of a triphenylethylene group, achieved a lower PCE of 5.5%. Although the reasons for this were not reported, this could be due to the reduced π-stacking imparted by the 3-dimensional triphenylethylene group compared with the planar naphthalene group. It was observed that the addition of 4-*tert*-butyl pyridine (TBP) to the electrolyte resulted in a poorer performance for the solar cell containing **16**.

**Figure 8:**
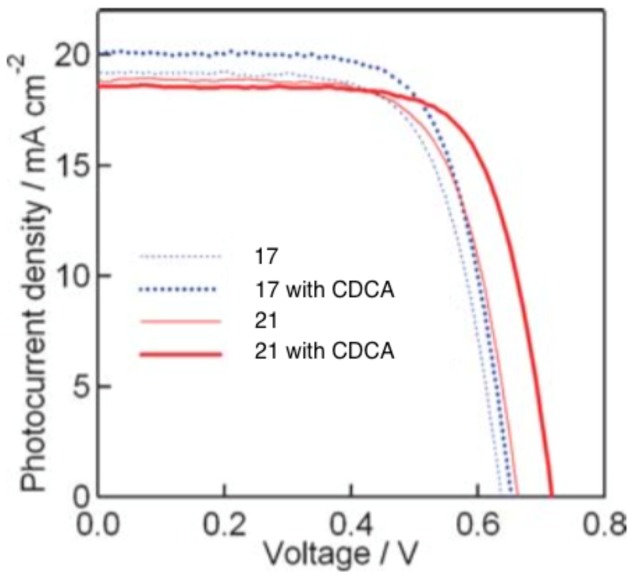
Current density vs. voltage characteristics for DSSCs with indoline dyes 17 and 21 as sensitizers with/without chenodeoxycholic acid (CDCA) under AM1.5 simulated sunlight (100 mW cm^−2^) illumination. (reprinted with permission from ref. [32], Copyright 2008 Royal Society of Chemistry).

Through the modification of the rhodanine portion of the dye, another series of dyes (**17**–**20**) was obtained [30]. The absorption of dyes **17**–**19** was red-shifted compared to dye **16** due to the incorporation of an additional rhodanine ring; and that of **20** was even further red-shifted as it contained three rhodanine rings. The extinction coefficient of dye **17** was 68,700 mol^−1^ cm^−1^ at 526 nm. Upon adsorption on TiO_2_, the absorption spectra were broadened, but there was no significant red-shift, indicating that the interaction between the dyes was reduced compared to those in **16**. Among this series of dyes, **17** delivered the best performance when used in DSSCs, with a PCE of 6.51%. Further optimization of the cells using chenodeoxycholic acid (CDCA) as an anti-aggregation coadsorbent improved the PCE to 8.00%. Through using an ionic-liquid-based electrolyte system and optimizing the thickness of the TiO_2_ electrode, an improved PCE of up to 9.03% was obtained [31]. To further control the aggregation between the dye molecules, the *n*-ethyl substituent on the rhodanine ring of **16** was replaced by an *n*-octyl substituent (**21**). Cells containing **21** delivered a PCE of up to 9.52% when they were processed using CDCA as an additive (Figure 8) [32].

A further example of rotor dyes in DSSC was reported by Li and co-workers [33]. Two tetraphenylethylene (TPE) moieties were attached to a triphenylamine group, which was then linked to the cyanoacrylic acid group through thiophene or furan linkers (**22**–**23**). In comparison to their parent dyes without TPE moieties, the absorbance of dyes **22** and **23** are slightly red-shifted. This could be attributed to the extension of the π-conjugated system upon incorporation of the TPE. The molar extinction coefficients of the dyes were over 30,000 mol^−1^ cm^−1^, which are higher than those of most ruthenium dyes. When applied in DSSCs, both dyes delivered approximately the same PCE of ca. 5.6%. However the addition of CDCA improved the performance of **23** significantly to 6.77% as a result of an improvement in both *J_sc_* and *V_oc_*, while that of **22** only increased slightly to 5.87% as the *V_oc_* improved but not the *J_sc_*. These results indicate that the dye aggregation remains a problem in these devices. To further enhance the *anti*-aggregation properties of the dyes, Chen and co-workers incorporated triphenylethylene phenothiazine and triphenylethylene carbazole moieties into dye molecules, forming a D-D-π-A structure (**24**–**27**) [34]. In addition to aggregation control, the incorporation of the triphenylethylene structure was expected to extend the absorbance range, improve hole transport, and tune the energy levels of the frontier orbitals. The role of the phenothiazine and carbazole moieties was to improve the hole transport properties of the dye. Upon comparing the absorbance spectra of the dyes, the triphenylethylene phenothiazine moiety was discovered to have better absorption than the triphenylethylene carbazole moiety. When applied in DSSCs, **24** delivered a PCE of 2.14%. The replacement of the planar carbazole moieties with twisted phenothiazine moieties improved the performance of the dyes. Dye **24**, which had three phenothiazine moieties, achieved the highest PCE of 6.55%. The introduction of the non-planar triphenylethylene structure into the dye structure resulted in an increase in the *V_oc_* values compared to their parent dyes, which may be due to the suppression of charge recombination in the non-planar structures [26] as well as a reduction in back-electron transfer arising from reduced dye aggregation. This group also systematically introduced polyphenyl-substituted ethylene end groups onto a phenothiazine dye (**28**–**30**) [35]. Of the three end groups (diphenylethylene, triphenylethylene, and tetraphenylethylene), the latter two were considered AIE active, while dyes **24**–**27**, exhibited ACQ. The PCEs of DSSCs based on these dyes were between 5.79% and 6.29%, with **29** delivering the best performance. The *V_oc_* was observed to increase with increasing numbers of phenyl rings on the end-capping group. Analysis of the dark current showed that the dark current decreased with increasing numbers of phenyl rings on the end-capping group, implying that the improved *V_oc _* was associated with reducing the recombination of injected electrons. The authors used intensity modulated photovoltage spectroscopy (IMVS) and intensity modulated photocurrent spectroscopy (IMPS) to show that electron transport at high light intensity may be affected by the incorporation of a highly rigid end group (**30**).

## 5. Organic Photovoltaics

**Figure 9:**
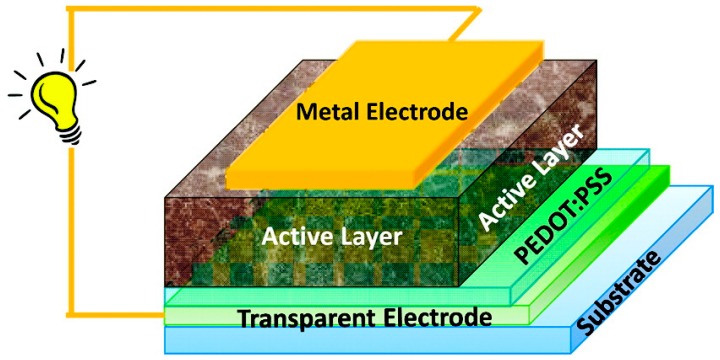
Structure of a typical bulk-heterojunction organic photovoltaic (OPV) cell. (reprinted from ref. [40] with permission. Copyright 2012 American Chemical Society).

Like DSSCs, OPVs have the potential to be manufactured in large scale via roll-to-roll printing techniques, thereby enabling the low-cost harvesting of solar power [36]. In an OPV cell, the active material is a blend of two materials—an electron donor and an electron acceptor. These materials form a bulk heterojunction (BHJ) which is an interpenetrating network that facilitates efficient charge separation (Figure 9). Many donor and acceptor materials have been developed in recent years, and these materials have played a major role in pushing the PCEs of these cells past the 10% mark [37,38,39]. Although there was an early report on donor molecules containing the AIE-active hexaphenylsilole moiety, the major impact of rotor-containing molecules has been on non-fullerene acceptors, where the rotors are used to control aggregation and improve electron transport.

### 5.1. Donors

**Figure 10:**
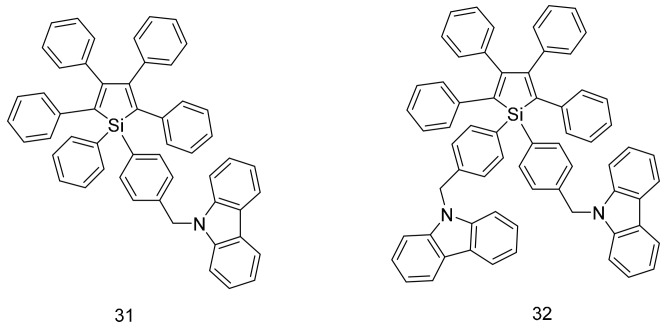
Structures of hexaphenylsilole molecules used as donors in OPV.

The use of molecules with rotor structures in OPVs was first reported in 2005. By attaching electron donating carbazolyl groups to an AIE-active hexaphenylsilole core, photoactive donor-acceptor adducts **31** and **32** were achieved (Figure 10) [41]. The thermal behavior of these molecules were investigated using thermogravimetric analysis and differential scanning calorimetry, and it was determined that the addition of the carbazolyl groups helped to increase the thermal and morphological stability of the molecules compared to the hexaphenylsilole core. Optical studies revealed that both molecules form nanoaggregates when large amounts of water (92%) are added to their acetone solutions. The emission quantum yield of **32** was significantly less than that of **31**, suggesting that that there was some degree of charge dissociation in **32**. This was confirmed by the performance of **32** when it was applied in OPV cells, with power conversion efficiencies (PCEs) reaching up to 2.19% for a device with the structure ITO/NPB(200 Å)/Alq3:5% **32** (300 Å)/Alq3 (50 Å)/LiF(7 Å)/Al. 

### 5.2. Acceptors

For a long period, fullerene derivatives such as [6,6]-phenyl-C_61_-butyric acid methyl ester (PCBM) were ubiquitous as the acceptor material in high performance OPVs. These materials offer advantages such as high (and relatively isotropic) electron mobility and the ability to form domains that are small enough to facilitate efficient charge separation. However, they also suffer from drawbacks such as poor absorption and high production cost. In recent years, acceptor materials based on non-fullerene small molecules have been developed [10]. A common limitation of these small molecule acceptors is their tendency to aggregate into large domains in BHJ blend films. To counter the aggregation, small molecules with twisted structures or with bulky bridging groups have been designed, resulting in acceptors which are able to form amorphous films with acceptably small domain sizes; however the electron mobilities of these materials are insufficient to deliver high OPV performance. 

The unique properties of rotor structures may be useful in designing non-fullerene acceptor molecules as their highly-twisted structures result in weak inter-molecular interactions and good solubility in organic solvents. Yan and co-workers designed and prepared a 3-dimensional molecule with four perylenediimide (PDI) moieties attached to a TPE core (**33**) (Table 3) [42]. This molecule exhibited excellent solubility in common organic solvents, even in hexane, hence it could easily be fabricated into thin films using solution processing techniques such as spin-coating. X-ray diffraction analysis revealed that the films were amorphous, in contrast to the highly crystalline films that are typical for PDI-based molecules. The TPE core was thus shown to be effective in suppressing the aggregation of the PDI moieties.

**Figure 11:**
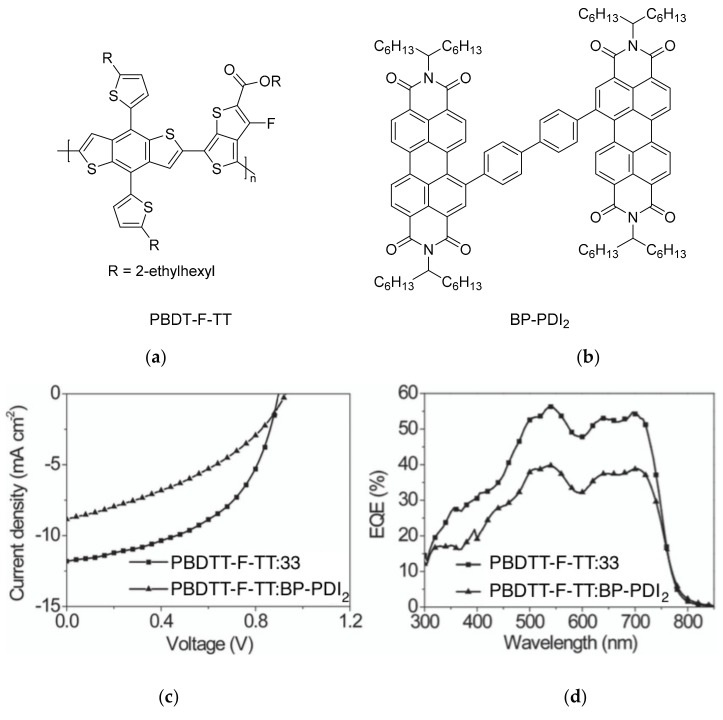
(**a**) Chemical structure of polymer donor PBDTT-F-TT; (**b**) Chemical structure of biphenyl-bridged perylene diimide dimer (BP-PDI_2)_; (**c**) current-voltage characteristics of bulk heterojunction (BHJ) solar cells fabricated from PBDTT-F-TT:33 and PBDTT-F-TT:BP-PDI_2_ under AM1.5 irradiation at 100 mW cm^−2^; (**d**) External quantum efficiency spectra of the devices. (reprinted with permission from ref. [42]. Copyright 2015 Wiley-VCH).

BHJ OPV devices were fabricated from blend films of **33** with the donor polymer PBDTT-F-TT (Figure 11), with the inverted structure of ITO/ZnO/PBDTT-F-TT:**33**/V_2_O_5 _(20nm)/Al (100 nm). Atomic force microscopy of the blend films revealed surface features on the order of 20–30 nm, showing that **33** does not form large domains within the blend film. Promising PCEs of up to 5.53% were obtained. Notably, the *V_oc_* of 0.91 V was significantly higher than PBDTT-F-TT:PCBM cells. This could be attributed to the twisted structure of the TPE core, which resulted in negligible conjugation between the PDI units as confirmed by cyclic voltammetry and UV-Vis measurements.

The external quantum efficiency of the cells exhibited a peak at 540 nm, which corresponded with the absorbance peak for **33**, indicating that unlike PCBM-based cells, the acceptor contributed significantly to the light absorption of the blend film. The 3D structure of **33** was also demonstrated to result in a higher electron mobility (1 × 10^−3^ cm^2^ V^−1^ s^−1^) than BP-PDI_2_ (2 × 10^−4^ cm^2^ V^−1^ s^−1^) through space-charge-limited current measurements. 

**Figure 12:**
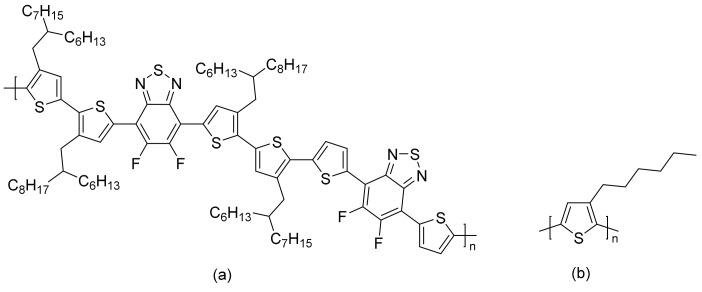
Structures of donor polymers (**a**) PffBT-T3; (**b**) poly(3-hexylthiophene) (P3HT).

While intramolecular twisting is an effective tool in the design of small molecule acceptors, the degree of intramolecular twisting may need to be optimized. In this regard, Yan and co-workers designed **34**, which had another rotor structure, tetraphenylpyrazine (TPPz), at its core [43]. As the TPPz core was larger and less twisted than TPE, the degree of intramolecular twisting in **34** was less than that of **33**. This was immediately evident in the reduced solubility of **34** compared in chlorobenzene with that of **33**. Using the space-charge-limited current method, the electron mobility of **34** was estimated to be 2.3 × 10^−3^ cm^2^ V^−1^ s^−1^, significantly higher than that of **33**. UV-Vis data also showed that there was a blue-shift in the main absorption peak of **34** going from solution to film, which may be attributed to the formation of *h*-aggregates. Inverted OPV cells, with the structure ITO/ZnO/ PffBT-T3:**34**/MoO_3_/Al, utilizing PffBT-T3 (Figure 12) with **34** delivered high PCEs of up to 7.1%, with the best performing cells having an excellent *V_oc_* of 0.99 V. Despite having a stronger aggregation tendency than **33**, the domain sizes of **34** in PffBT-T3:**34** blend films were still reasonably small when observed under atomic force microscopy. This observation was supported by photoluminescence quenching studies which revealed that the charge transfer process in the PffBT-T3:**34** blend films was highly efficient.

Bhosale and co-workers reported a TPE-core acceptor using diketopyrrolopyrrole (DPP) units in place of PDI units (**35**) [44]. This molecule exhibited excellent solubility in *o*-dichlorobenzene. Cells with the structure of ITO/PEDOT:PSS (38 nm)/poly(3-hexylthiophene):**35**/Ca (20 nm)/Al (100 nm) gave PCEs of up to 3.86%, with *V_oc_* of 1.18 V. This *V_oc_* was among the highest reported for single junction BHJ OPV devices. For comparison, a P3HT:PC_61_BM fabricated using similar conditions yielded PCEs of up to 2.85%. It is possible that combining **35** with higher-performance donor polymers would lead to higher PCEs than what had been obtained with P3HT.

Liu and co-workers reported a similar molecule, **36**, which had an additional phenyl ring attached to each DPP arm [45]. The molecule was synthesized using sequential direct C-H arylation with a high overall yield. The authors of this study used poly [(9,9-*bis*(30-(*N*,*N*-dimethylamino)propyl)-2,7-fluorene)-alt-2.7-(9,9-dioctylfluorene)] (PFN) as a cathode buffer layer, resulting in a device structure of ITO/PEDOT:PSS/P3HT:**36**/PFN (5 nm)/Al (100 nm). *V_oc_* of up to 1.18 V was obtained; however the relatively poor *J_sc_* of 4.55 mA/cm^2^ and FF of 0.47 resulted in the cells having PCEs of only up to 2.49%.

## 6. Perspective & Conclusions

Since their first discovery, remarkable progress has been made on fluorogens with rotor structures. Although there have been relatively few examples of rotor structures in solar cell applications, the results have been highly promising. In solar cell research, rotor structures have been used to create optically active molecules with large Stokes shifts and suppressed π-stacking for luminescent down-shifting devices and luminescent solar concentrators. The improved properties of these rotor molecules have helped to address parasitic reabsorption losses and fluorescence quenching in the solid state. Further improvements in LDS and LSC devices may be obtained by optimizing the photophysical properties of the AIEgens, such as absorption and emission wavelengths, to better match the characteristics of the solar cells.

In third-generation solar cells, rotor structures have also been incorporated into metal-free dyes for dye-sensitized solar cells and non-fullerene acceptors in organic photovoltaics. The aggregation control provided by these rotors have resulted in improved solar cell PCEs, and may enable these materials to be competitive with their more expensive counterparts, namely ruthenium-based dyes and fullerene derivatives, respectively. Continued optimization of molecules with rotor structures may drive further improvements in solar cell efficiencies and facilitate cost reductions. Particularly, other rotor structures with different degrees of twisting may be explored. Substituents such as alkyl or aryl groups may also be introduced onto the rotor structures to tweak the three-directional molecular conformation of the molecules. Further research into the unique twisted structures and photophysics of rotor structures may also open up new applications in solar cell research beyond the ones that have already been reported. Together with progress in other aspects of solar cells, such as device architectures, materials, and encapsulation, rotor-containing fluorogens may yet make a significant contribution towards a brighter future.

## Figures and Tables

**Table 1:** Structures and optical properties of fluorophores with Aggregation-Induced Emission properties (AIEgens) used in Luminescent Down-Shifting (LDS) devices.

No.	Structure	λ_abs_ (nm)	λ_em_ (nm)	*Δ*λ (nm)	*Φ_f_*	Ref.
**1**	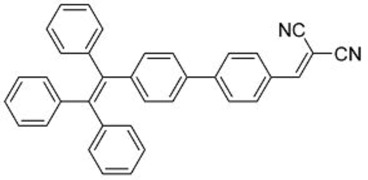	388	534	146	0.99	[17]
**2**	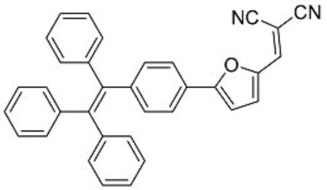	433	562	129	0.93	[17]
**3**	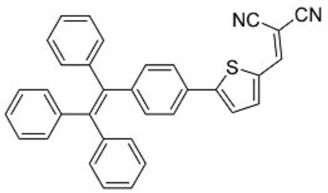	431	574	143	0.84	[17]
**4**	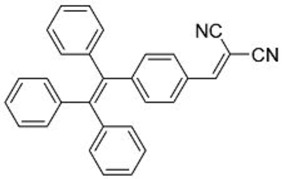	392	532	140	0.99	[18]
**5**	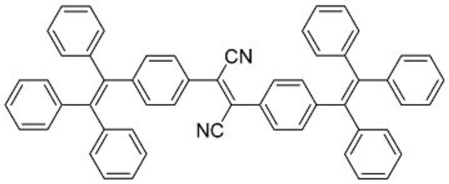	406	576	170	0.98	[18]
**6**	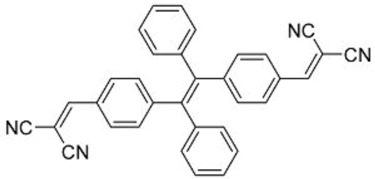	397	533	139	0.81	[18]
**7**	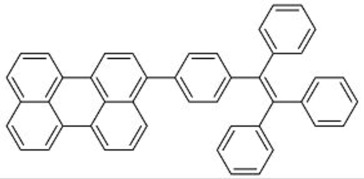	448	512	64	1.00	[19]

**Table 2:** Examples of metal-free DSSC dyes containing rotor structures. All examples below utilize liquid (I^−^/I_3_^−^) electrolyte solutions.

No.	Structure	*J_sc_* (mA/cm^2^)	*V_oc_* (V)	Fill Factor	PCE (%)	Ref.
**16**	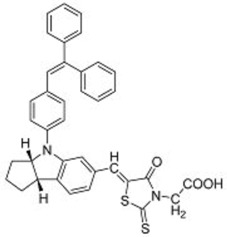	18.07	0.559	0.55	5.5	[29]
**17**	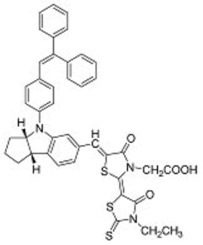	18.75 19.96	0.645 0.653	0.538 0.694	6.51 9.03	[30] [31]
**18**	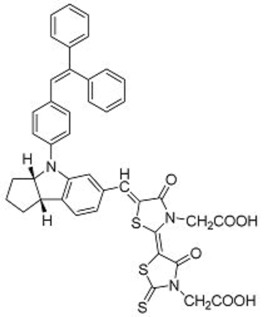	17.50	0.584	0.538	5.50	[30]
**19**	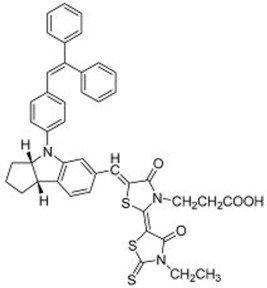	17.38	0.628	0.513	5.60	[30]
**20**	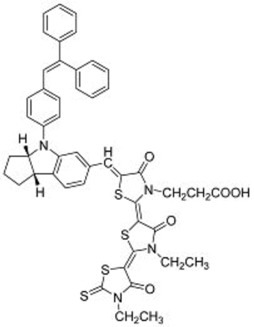	19.56	0.569	0.533	5.93	[30]
**21**	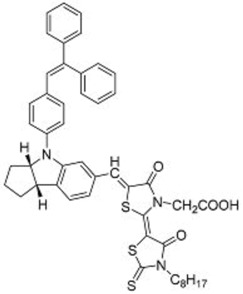	18.68	0.710	0.707	9.52	[32]
**22**	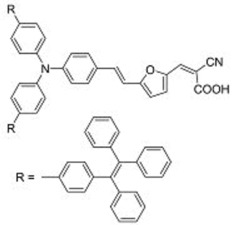	13.04	0.72	0.62	5.87	[33]
**23**	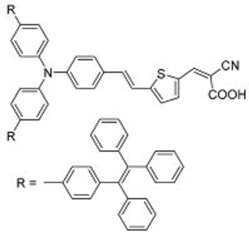	14.69	0.74	0.62	6.77	[33]
**24**	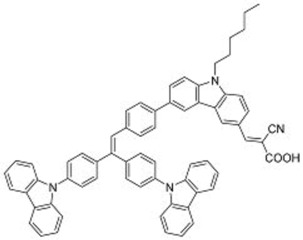	4.55	0.682	0.69	2.14	[34]
**25**	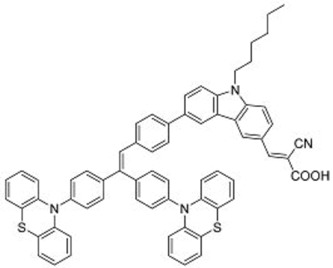	5.27	0.711	0.72	2.69	[34]
**26**	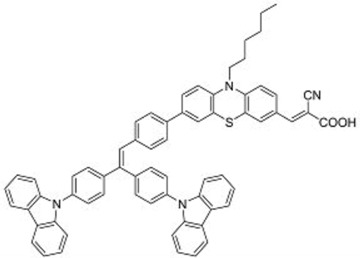	10.76	0.793	0.64	5.51	[34]
**27**	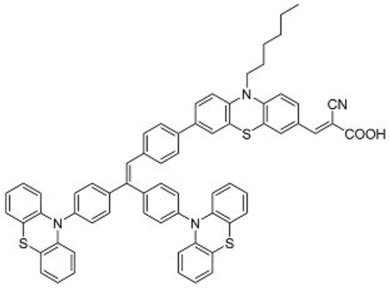	12.18	0.826	0.65	6.55	[34]
**28**	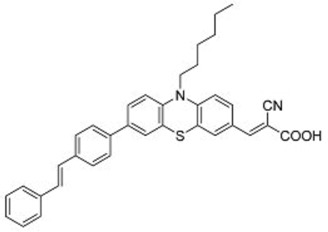	11.82	0.759	0.65	5.84	[35]
**29**	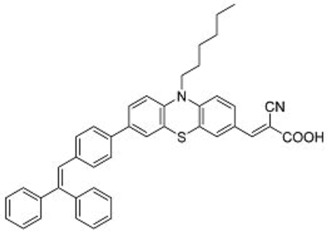	12.62	0.789	0.63	6.29	[35]
**30**	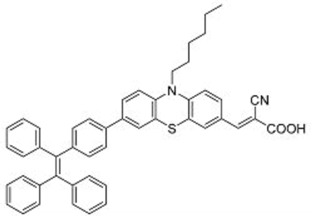	11.41	0.804	0.63	5.76	[35]

**Table 3:** Structures of OPV molecules containing AIE rotors.

No.	Structure	*J_sc_* (mA/cm^2^)	*V_oc_* (V)	Fill Factor	PCE (%)	Ref.
**33**	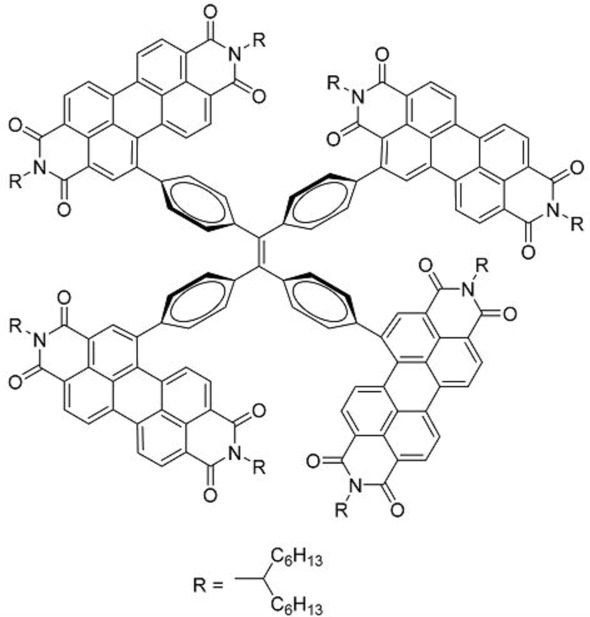	11.7	0.91	0.52	5.53	[42]
**34**	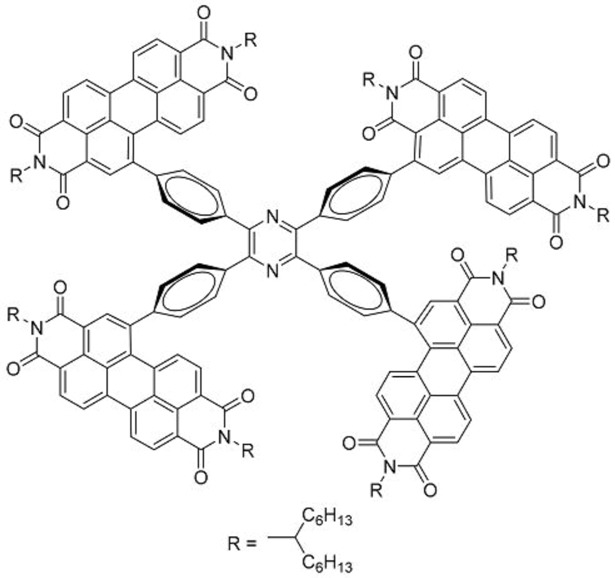	12.5	0.99	0.56	7.1	[43]
**35**	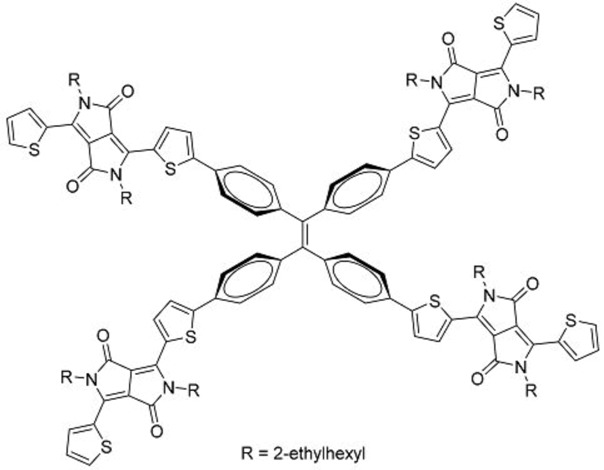	5.17	1.18	0.64	3.86	[44]
**36**	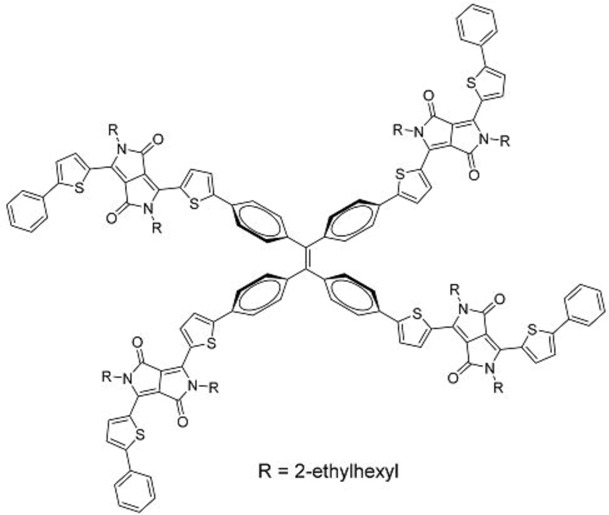	4.45	1.16	0.47	2.43	[45]
